# Relapse prevention therapy for internet gaming disorder in Swedish child and adolescent psychiatric clinics: a randomized controlled trial

**DOI:** 10.3389/fpsyt.2023.1256413

**Published:** 2023-10-20

**Authors:** Frida André, Sabina Kapetanovic, Isak Einarsson, Sunna Trebbin Harvard, Leonard Franzén, Annika Möttus, Anders Håkansson, Emma Claesdotter-Knutsson

**Affiliations:** ^1^Department of Clinical Sciences, Lund, Faculty of Medicine, Lund University, Lund, Sweden; ^2^Department of Social and Behavioral Sciences, University West, Trollhättan, Sweden; ^3^Region Skane, Child and Adolescent Psychiatry, Regional Outpatient Care, Lund University Hospital, Lund, Sweden; ^4^Civic Centre Children and Youth, The Social Services Administration, Copenhagen, Denmark; ^5^Social Services, Malmö, Sweden; ^6^Region Skåne, Malmö Addiction Centre, Gambling Disorder Unit, Malmö, Sweden

**Keywords:** gaming, internet gaming disorder, CBT, GASA, relapse prevention

## Abstract

**Objectives:**

To evaluate the effectiveness of relapse prevention (RP) as a treatment for internet gaming disorder (IGD).

**Design:**

Randomized controlled trial.

**Setting:**

Three child and adolescent psychiatry (CAP) units in Region Skåne, Sweden.

**Participants:**

Children aged 13–18 years, coming for their first visit to CAP during 2022, were screened for gaming behavior. Those who met the proposed DSM-5 criteria for IGD were offered participation in the trial, if they had the capacity to provide written informed consent and if they spoke Swedish. A total of 111 CAP patients agreed to participate. Out of those, 11 patients were excluded due to incorrect inclusion such as young age (*n* = 1), or due to the absence of responses to follow-up measures (*n* = 9). After exclusion, 102 participants remained (intervention = 47, control = 55).

**Interventions:**

The intervention, RP, is based on cognitive behavioral treatment (CBT) and was provided individually, comprising of five to seven 45-min sessions over a period of 5 to 7 weeks versus treatment as usual.

**Outcome measures:**

Participants were assessed with Game Addiction Scale for Adolescents pre-treatment (GASA) (baseline), post-treatment (treatment group only), and 3 months after baseline (follow-up).

**Results:**

The repeated measures ANOVA showed a significant interaction effect between treatment and time. Both the control group and treatment group lowered their mean GASA score from baseline to follow-up significantly, but the improvement was greater in the treatment group (mean difference in control group −5.1, *p* < 0.001, 95% CI = − 3.390 to −6.755, mean difference in treatment group −9.9, *p* < 0.001, 95% CI = −11.746 to −8.105).

**Conclusion:**

RP was found to be superior to treatment as usual in terms of reduction of IGD symptoms. Future research should address which aspects within a given treatment are effective, who benefits from treatment, in what aspects, and why.

**Trial registration number:**

ClinicalTrials.gov, NCT05506384 https://clinicaltrials.gov/ct2/show/NCT05506384.

## 1. Introduction

Gaming is one of the most common leisure activities among children and adolescents and is nothing more than a source of entertainment, for the majority. However, some individuals engage in gaming in a way, and to such an extent, that negative consequences ensue (1–3). For some, gaming activity can become so extensive and severe that other activities and obligations, such as school, social relationships, and even physical needs, are neglected (2, 4). Most research agrees on the pathological potential of the behavior which has reached formal recognition with inclusion in both the *Diagnostic and Statistical Manual of Mental Disorders* (DSM-5) and in the *International Classification of Diseases* (ICD-11). Gaming disorder (GD) has its own diagnostic code in ICD-11 while the DSM-5 mentions internet gaming disorder (IGD) as a tentative diagnosis requiring more clinical research (5, 6). The DSM-5 definition of IGD is similar to their definition of pathological gambling, and so is most of the numerous existing screening tools (6–8).

Despite the increasing amount of research on IGD, controversy remains regarding fundamentals such as the validity of the condition but also regarding terminology, measurement approach, and diagnostic cut-off (7–9). The greatly varying estimates of prevalence and comorbidity are likely influenced by the controversies and discord. The reported prevalence of IGD varies across studies but has globally been estimated as approximately 3%, with the highest numbers found in adolescent samples (8). Apart from age, male gender is an established risk factor, and commonly listed comorbidities are ADHD, anxiety, and depression (1, 10). IGD is further known to cause impairment in both school performances and sleep habits – causing great concern in child and adolescent psychiatry (CAP) and school healthcare (2, 11, 12).

There is no consensus on how to treat IGD, over the past years, a few treatment studies have been published (13). However, these studies have been criticized for poor design and methodological flaws such as lack of control groups (13–15). Cognitive behavioral treatment (CBT) is one of the few methods that have been explored in relation to IGD (13, 14) and is recommended as a first line of treatment (16).

Relapse prevention (RP) is a CBT-based treatment developed to treat alcohol problems in adults, but the method is also used to treat addiction to alcohol, drugs, tobacco, and gambling among both adults and adolescents (17). RP focuses on cognitive restructuring, control of, and recognition of triggers for a problem behavior and the method has been raised as a possible therapy for IGD (18). RP is a relatively short and low-cost treatment which is also an established and well-received treatment method within the clinics that are part of the current project. We developed a CBT-based manual derived from RP for treatment of child and adolescent IGD. Together with experienced clinical psychologists, the manual was adjusted to suit children and adolescents within the CAP context. The number of sessions was reduced, and a fictionalized person was incorporated in a series of vignettes when demonstrating a particular theme. In a pilot study, we evaluated RP as a treatment for IGD and gambling among children and adolescents, showing promising results (19).

While most youth engage in gaming to some extent, a minority need help to control their gaming or to reduce the negative consequences thereof. To this date, no specific treatment is offered to children and adolescents suffering from IGD. Given this, our aim was to evaluate the effectiveness of RP as a treatment for problematic gaming within a CAP setting.

## 2. Methods

### 2.1. Trial design and setting

The current study is a non-blinded randomized control trial, performed within three different child and adolescent psychiatric (CAP) units in Region Skåne, Sweden. Detailed methods are described in the trial protocol paper (20).

In our protocol, we specified that our aim in this trial was to determine the effectiveness of RP as a treatment of not only IGD but also problem gambling (20). The results regarding gambling will be published separately.

### 2.2. Ethics approval

The study was reviewed and approved by the Swedish Ethical Review Authority (Ref 2019-04797, December 13, 2019). Subsequent amendments have been approved (Ref 2021-05592-01, January 3, 2021; Ref 2022-01289-02, March 15, 2022).

### 2.3. Participants

**Figure 1:**
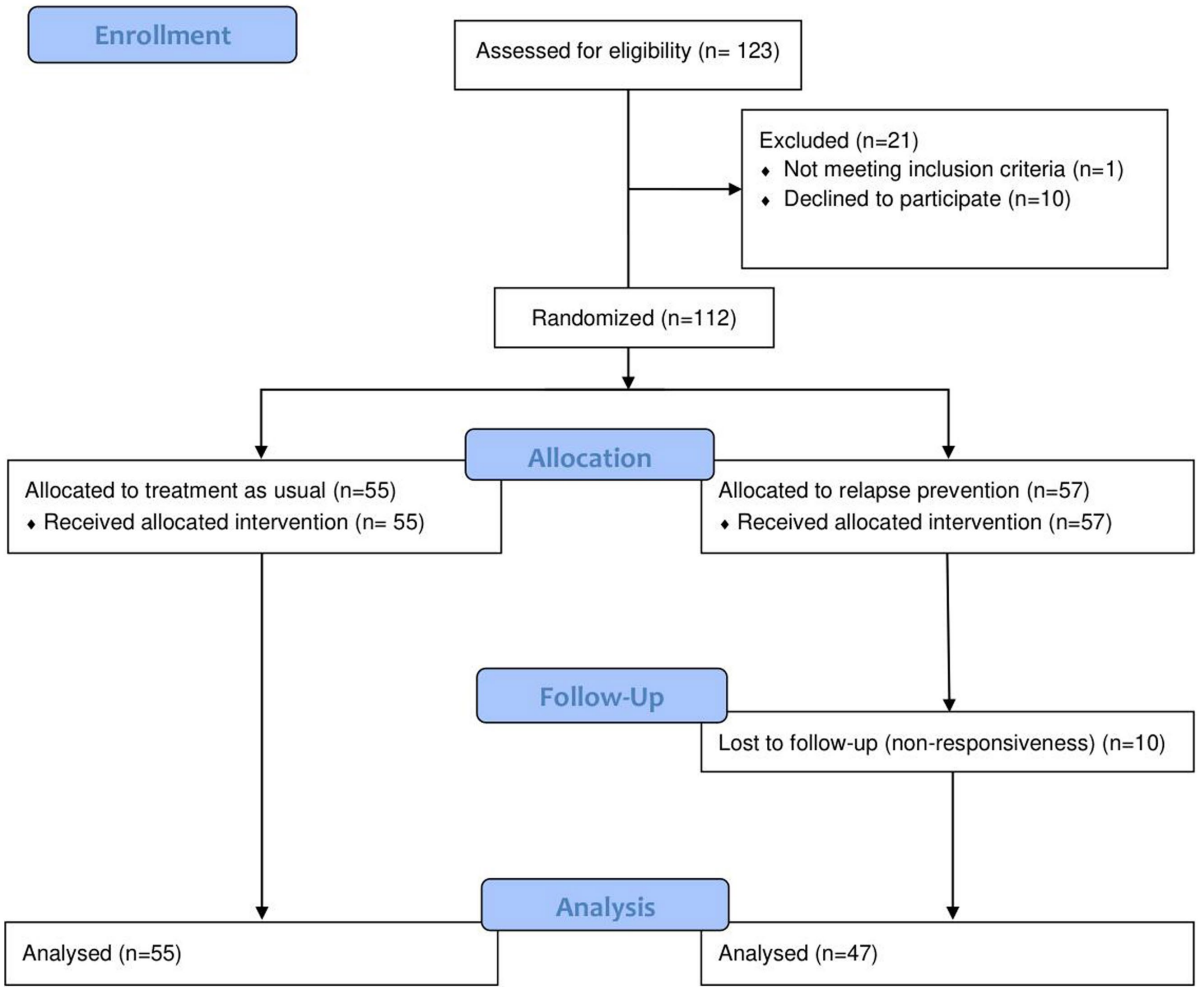
Flow diagram. Inclusion, exclusion, and randomization.

This trial and recruitment were performed from 1 September 2021 to 30 December 2022. Due to administrative error the trial was not registered in the clinicaltrials.gov until August 2022. All patients, between the years 13–18, coming for their first visit to CAP, were supposed to be screened via an application called The Blue App, for gaming behavior. Those meeting the proposed DSM-5 criteria for IGD (6) were offered participation in the trial, if they had the capacity to provide written informed consent and if they spoke Swedish. Unfortunately, not every patient was screened digitally due to technical problems, thus some were provided the assessment on paper. Caregivers’ consents were required for children younger than 15 years. Out of 2,630 new visits, we were able to register 622 (≈24%) patients assessed with GASA whereof 123 (≈20%) met the cut off for IGD. In the study protocol for this trial, we present a power calculation estimating that approximately 40% in the intervention group and 20% in the control group would improve by follow-up. With these figures, we estimated that 160 (80 + 80) patients should be included in the trial for us to be able to demonstrate a significant difference with sufficient power (20). However, among the CAP patients meeting the criteria for IGD during the study’s inclusion period, a total of 113 patients agreed to participate. One patient was excluded due to incorrect inclusion, being younger than 13 years old, and 10 patients were excluded because of not completing follow-up measures. The final sample consisted of 102 participants aged between 13 and 18 years old (M age = 14.42 years, SD = 1.367). For an overview of the inclusion, exclusion and randomization, see the flow diagram in Figure 1.

### 2.4. Randomization

Participants were randomized in a 1:1 ratio to either intervention or control. For randomization, we applied a random allocation sequence using the ‘chit method’ by preparing 160 chits of paper indicating either control or treatment (21). Each patient was distributed to a condition (control or treatment), and the chit was not replaced if the patient dropped out of the study. The control group received treatment as usual (TAU) at their home clinic. It was not possible to blind either participants, clinicians, or supervising researchers to randomization allocation.

### 2.5. Intervention

We collected pre-intervention (baseline) data from the participants before starting treatment. The treatment ran for 5 to 7 weeks for each participant. Post-intervention data were collected at weeks five to seven after completion of treatment. Follow-up data were collected 3 months after baseline date. The intervention ran for 14 months in total with final data collection and closure in month 16. We planned for the treatment to consist of seven to nine sessions over a period of 7 to 9 weeks. Based on experience from our pilot study (19), we decided to compress the treatment to facilitate participation. Consequently, the number of sessions differs from our protocol (20). The participants were considered dropouts if they completed less than five sessions.

#### 2.5.1. Relapse prevention

Participants assigned to the treatment group were administered RP over the course of five to seven sessions, each session lasting 45 min. The intervention was provided individually at the respective CAP units or via video link and was led by a clinician. The clinicians implementing the treatment were four licensed psychologists, certified in accordance with the Swedish National Board of Health and Welfare, one social worker, and one psychiatrist; all of them had competence in CBT. The treatment consists of three parts: (1) setting goals, in which the clinician examines the patient’s unwanted behavior, mapping his/her motivation for change and goals with treatment; (2) understanding and identifying high-risk situations and problem behaviors; and (3) identifying future high-risk behaviors and early warning signals and consolidating the new activity schedule. An important part of the treatment was theme- specific homework given at the end of each session to be discussed and evaluated at the next.

#### 2.5.2. Treatment as usual

Neither CAP, school healthcare staff, nor social services currently provide any treatment to children and adolescents who need help to stop or regulate their gaming behavior. Consequently, participants in the control group who received TAU received different interventions according to existing practice. Treatments provided in the control group were counseling (*n* = 21), medication for ADHD (Methylphenidate *n* = 22, Dexamphetamine = 1), antidepressants (Sertraline *n* = 1), referral to other unit (*n* = 1), further psychiatric evaluation (*n* = 1). Some individuals (*n* = 3) were put on a waiting list and did not start treatment, and some (*n* = 2) were discharged from CAP during the study period.

### 2.6. Measures

In addition to assessment regarding gaming behavior, basic demographics routinely recorded in the journal, such as gender, age, housing situations, and diagnosis, were collected. The treatment group was assessed with GASA regarding gaming (22) at baseline (before treatment), after the treatment, and at follow-up (3 months after baseline assessment). The control group were assessed with GASA at baseline and at follow-up.

#### 2.6.1. GASA

The 7-item GASA was used to screen for IGD (22). GASA is one of the most frequently used measures for IGD (22, 23). The instrument is based on the DSM criteria for problem gambling (salience, tolerance, mood modification, relapse, withdrawal, conflicts, and problems) and applies to gaming behavior during the past 6 months (22). The DSM suggests that half of the criteria should be met to qualify for a diagnosis. However, a ranking of the constituent items has been proposed. It has been argued that the ‘core criteria’ of relapse, withdrawal, conflicts, and problems relate more heavily to addiction than the criteria that concern salience, tolerance, and mood modification, which, according to some scholars, should be considered peripheral (16, 24, 25). Therefore, the ‘core approach’ applies a prioritization of the four core criterion, creating three categories of gamers: engaged gamers, problem gamers, and addicted gamers. This approach has been reported as clinically relevant as the created categories seem to relate to degrees of negative consequences as well as severity of addictive behavior (25, 26).

Responses were given on a 5-point scale from 1 = never, to 5 = very often. An item was considered endorsed when rated 3 or higher (22). The scale produces two outcome measures: firstly, a continuous GASA score with a minimum of seven points to a maximum of 35 and secondly, categories of gamers (engaged, problem, and addicted gamers) in accordance with the core approach (24).

### 2.7. Data preparation

Statistical analyses were performed in SPSS (IBM SPSS statistics version 27). Gender, housing situation, and diagnosis were recoded into binary variables (Yes = 1/No = 0). The least prevalent diagnoses were merged into a new variable labeled ‘other diagnosis’ (see Table 1). This variable included anxiety disorders (anxiety disorder, unspecified, ‘mixed anxiety, and depressive disorder, generalized anxiety disorder), other symptoms and signs involving emotional state, obsessive compulsive disorder, adjustment disorder, pathological gambling, and diagnoses primarily used during the psychiatric evaluation phase (observation for suspected mental and behavioral disorders, general psychiatric examination, not elsewhere classified, examination and observation for unspecified reason, observation following alleged rape or seduction, examination and observation for unspecified reason).

**Table 1:** Sample characteristics.

	Control		Treatment		Total	
	Frequency	%	Frequency	%	Frequency	%
Total sample	55	53.9	47	46.1	102	100
Dropouts	0	0	6	5.7	6	5.9
Gender						
Male	36	65.5	39	83.0	75	73.5
Female	19	34.5	8	17.0	27	26.5
Age, years						
13–15	43	78.2	38	80.9	81	79.4
16–18	12	21.8	9	19.1	21	20.6
Housing situation						
Cohabiting parents	33	60.0	23	48.9	56	54.9
Separated parents	22	40.0	24	51.1	46	45.1
Diagnosis						
ADHD	20	36.4	17	36.2	37	36.3
ADD	10	18.2	3	6.4	13	12.7
ASD	6	10.9	5	10.6	11	10.8
Depression	2	3.6	5	10.6	7	6.9
Other diagnosis	17	30.9	17	36.2	34	33.3

The sum of GASA score at baseline, after treatment, and at follow-up composed separate continuous variables used as outcome measures for ANOVA analysis. The difference in score from baseline to follow up, labeled ‘improvement’, constituted another continuous outcome variable used in a linear regression analysis.

Individuals meeting every core criterion (16, 23–25) in GASA were categorized as ‘addicted gamers’. The respondents that endorsed two to three of the core criteria were categorized as problem gamers, and those who endorsed all three of the peripheral criteria but not more than one of the core criteria were categorized as ‘engaged gamers’. At follow-up, some participants did not meet the criteria for either of the gaming categories, and were labeled ‘<engaged gamers’.

### 2.8. Data analysis

The mean GASA score at baseline and at follow-up was used in a repeated measure ANOVA to compare the change in mean value between control group and treatment group. The treatment group was analyzed in a repeated measure ANOVA separately to compare the mean GASA score at baseline, after treatment, and at follow-up, against each other. The mean difference in GASA score between baseline and follow-up (improvement) was used in an independent sample *t*-test of the difference between treatment group and control group to unable an estimate of the effect size. The improvement in GASA score was also used as the dependent variable in a regression model to quantify the impact of treatment, with adjustment of baseline GASA score, demographics and comorbidity diagnosis.

McNemar’s test was applied to compare the prevalence of gaming categories between baseline and follow-up, in control group and treatment group separately.

## 3. Results

Sample characteristics are shown in Table 1. Out of the 102 participants, 46% constituted the treatment group, and 6% were dropouts. One-quarter of the total sample was female and constituted 17% of the treatment group and 30% of the control group. A majority were aged 13–15 years and the mean age was 14 years. The distribution of cohabiting and separated parents was relatively even. The most common diagnosis was ADHD followed by ADD, ASD, and depression.

At baseline, 11% met the cut off for engaged gaming in the control group and none in the treatment group. Problem gamers constituted 55 and 49% of the control and treatment group, respectively. Addicted gamers constituted 35 and 51% of the control and treatment group, respectively.

### 3.1. Reduction in mean GASA score

The following analyses were checked for assumptions of equal variance and normality, the assumptions were met.

**Figure 2:**
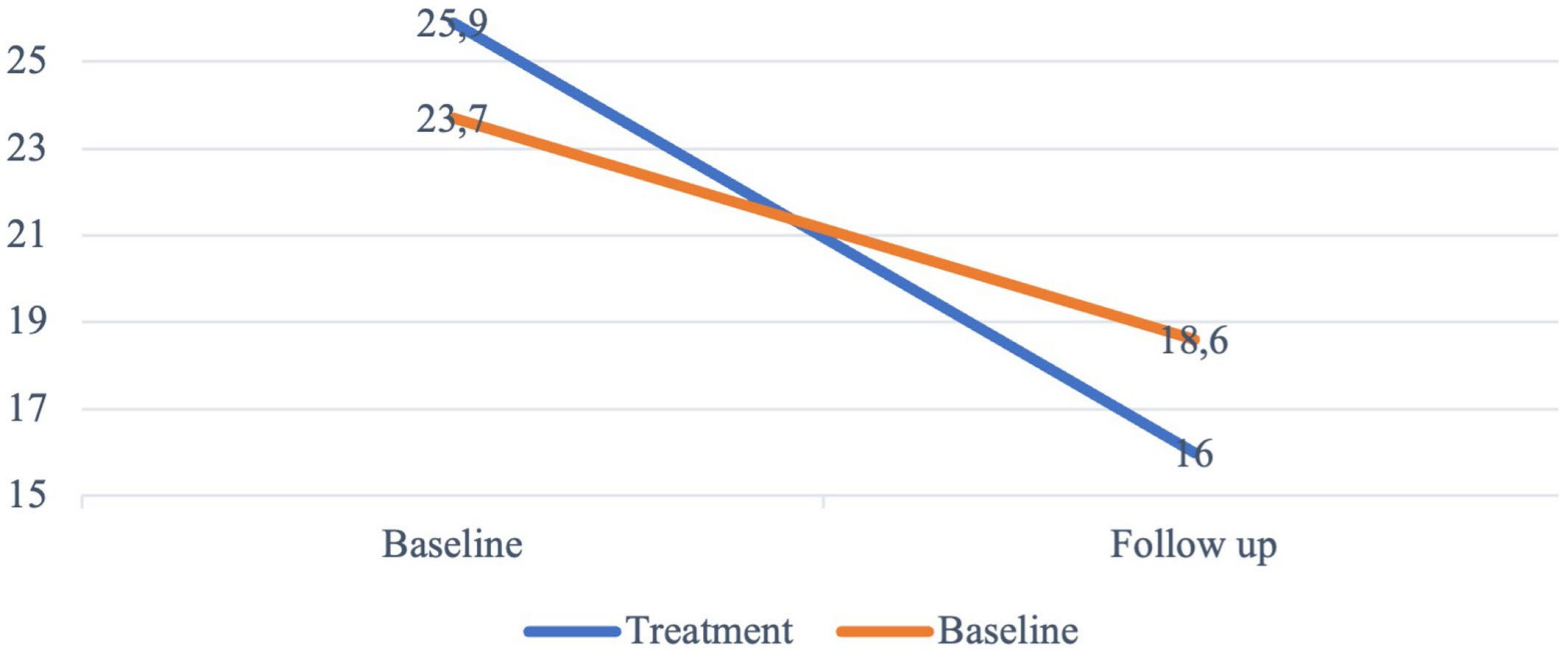
Mean GASA score. Changes in mean score from baseline to follow-up. *N* = 102.

As shown in Figure 2, both the control and treatment group lowered their GASA score over time. The repeated measures ANOVA test of within subject effects showed that there was a significant interaction effect between time and treatment (*p* < 0.001). The *post hoc* analysis of estimated marginal means (EMMEANS) showed that the mean GASA score differed significantly between control and treatment group, both at baseline (mean difference 2.2, *p* = 0.008, 95% CI = 0.578, 3.806) and at follow-up (mean difference −2.7, *p* = 0.026, 95% CI = −0.322, −4.999). Both the control group and treatment group lowered their mean GASA score from baseline to follow-up significantly (mean difference in control group −5.1, *p* < 0.001, 95% CI = − 3.390, −6.755, mean difference in treatment group −9.9, *p* < 0.001, 95% CI = −11.746, −8.105). The independent samples *t*-test showed a significant difference in the mean improvement in GASA scores between control group and treatment group (*t* = −3.88 (100), *p* = <0.001, CI = −7.331, −2.374). The effect size, as measured by Cohen’s d, was *d* = 0.77, indicating a medium effect (27).

The linear regression model is reported in Table 2. The regression analysis showed that the treatment contributed significantly to a greater difference in GASA score from baseline to follow-up, meaning that the improvement among those who underwent treatment was significantly greater. Additionally, the mean GASA score at baseline contributed significantly to the model; a high baseline score was positively associated to a greater improvement. Demographics, such as age, gender and housing situation, did not contribute significantly to any change in GASA score and neither did any of the most common diagnosis.

**Table 2:** Hierarchical linear regression analysis.

	Coefficients		Model summary			
Predictor	*β*	Sig.	*R* ^2^	Δ *R*^2^	ΔF	Sig. ΔF
Model 1			0.131	0.131	15.088	<0.001
Treatment	4.853	<0.001				
Model 2			0.277	0.146	19.995	<0.001
Treatment	3.472	0.004				
Baseline GASA score	0.630	<0.001				
Model 3			0.255	0.000	0.001	0.979
Treatment	3.468	0.005				
Baseline GASA score	0.629	<0.001				
Male gender	0.036	0.159				
Model 4			0.292	0.015	2.014	0.979
Treatment	3.514	0.004				
Baseline GASA score	0.639	<0.001				
Male gender	−0.050	0.970				
<Age 15	2.008	0.159				
Model 5			0.292	0.000	0.015	0.904
Treatment	3.501	0.005				
Baseline GASA score	0.637	<0.001				
Male gender	−0.038	0.978				
<Age 15	2.019	0.160				
Cohabiting parents	−0.142	0.904				
Model 6			0.292	0.025	0.832	0.508
Treatment	3.462	0.007				
Baseline GASA score	0.616	<0.001				
Male gender	0.080	0.953				
<Age 15	1.682	0.268				
Cohabiting parents	−0.003	0.998				
ADHD	1.348	0.355				
ADD	1.714	0.329				
ASD	1.999	0.111				
Depression	4.017	0.378				

Dependent variable GASA mean improvement.

**Figure 3:**
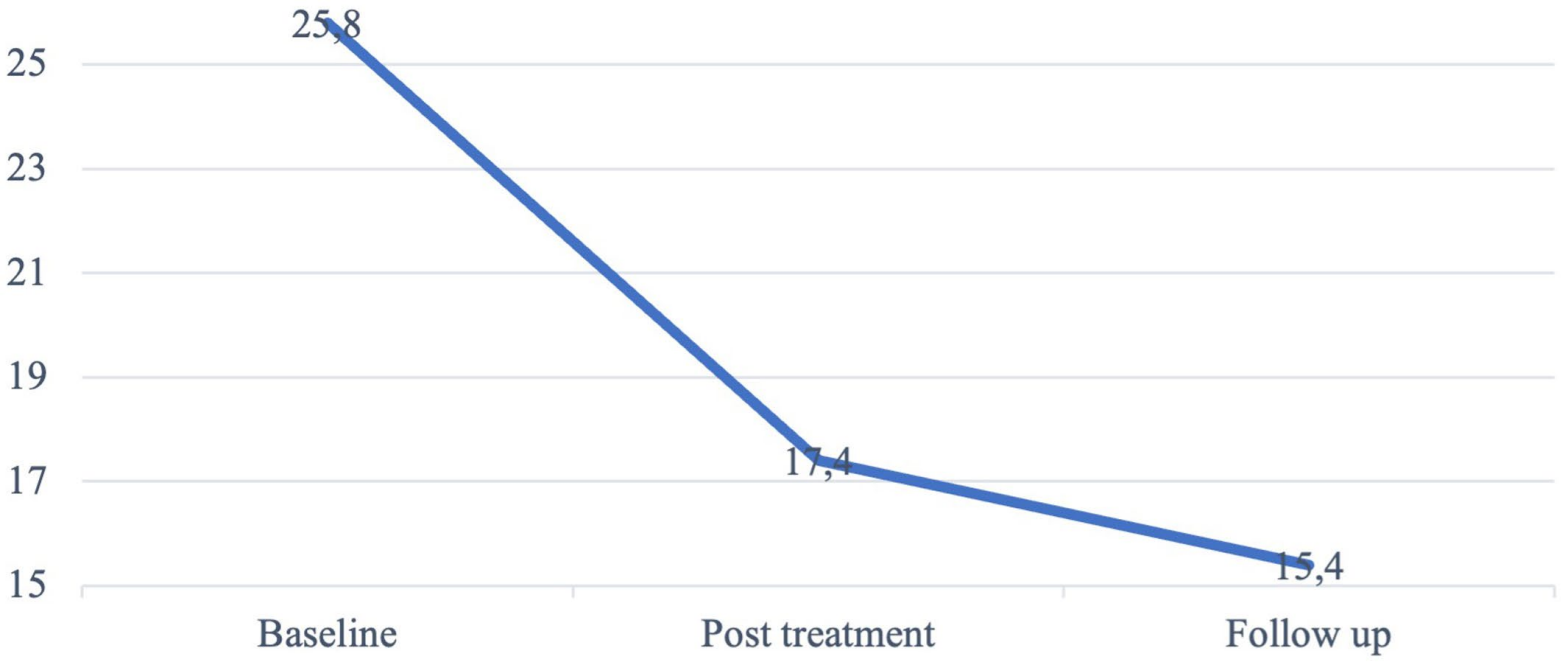
Mean GASA score at baseline, post-treatment, and at follow-up. Treatment group. *N* = 43.

The treatment group was further analyzed separately in a repeated measure ANOVA to unable incorporation of the GASA score collected immediately after treatment. The mean score from baseline, post treatment and follow-up are visualized in Figure 3. As the post-treatment GASA score was missing for five individuals, this analysis only included 43 participants. The mean difference in GASA score was significant, both between baseline and post-treatment (mean difference = 8.4, *p* < 0.001, 95% CI = −10.813 – −5.954), and from post-treatment to follow-up (mean difference = 2.0, *p* = 0.007, 95% CI = −3.612 – −0.481).

### 3.2. Reduction in gaming severity level

As shown in Table 3, McNemar’s test showed that the proportion of both problem and addicted gamers was significantly lower at follow-up in comparison to baseline in the treatment group whereas no difference was seen in the control group.

**Table 3:** McNemar’s test for *X*^2^ -comparisons of the prevalence of gaming categories between baseline and follow-up, in control group and treatment group separately.

	Control			Treatment		
	Baseline N (%)	Follow-up N (%)	*p*-value	Baseline N (%)	Follow-up N (%)	*p*-value
<Engaged gamers	0 (0)	34.5 (19)	–	0 (0)	59.6 (28)	–
Engaged gamers	10.9 (6)	5.5 (3)	0.453	0 (0)	4.3 (2)	–
Problem gamers	54.5 (30)	41.8 (23)	0.167	48.9 (23)	25.5 (12)	0.043
Addicted gamers	34.5 (19)	18.2 (10)	0.064	51.1 (24)	10.6 (5)	<0.001

*N* = 102.

## 4. Discussion

Interest in the treatment of IGD has clearly increased in recent years, from a basically non-existent level to an ever-increasing number of published articles on the subject (13, 14). It seems reasonable to assume that the interest in the treatment of IGD represents a need, identified by parents, school healthcare providers, and other caregivers seeing problems they interpret as related to excessive gaming among children. However, existing research within this field is still sparse and marked by methodological flaws (13).

The present RCT evaluates RP as a treatment for IGD among children and adolescents ages 13–18, within the context of CAP in southern Sweden. The participants were assessed regarding symptoms of IGD at baseline and at follow-up, carried out 3 months after the initial screening. In addition, the treatment group was also assessed regarding symptoms of IGD immediately after the treatment had been completed. Both the treatment group and the control group improved regarding IGD symptomatology from baseline to follow-up. In the treatment group, however, children and adolescents exhibited significantly greater improvement in terms of their IGD. Further, the proportion of both addicted and problem gamers showed a significant decrease from baseline to follow-up in the treatment group, whereas no difference was seen in the control group.

Relapse prevention was developed in the 80s, originally as a response to the failed long-term effects of other therapies at the time (17, 28). The method has ever since been used for various substance use disorders but also for the treatment of behavioral addictions and it has been suggested as a treatment for IGD specifically (23, 28). The treatment model aims to identify and address triggers or high-risk situations/circumstances in order to prevent relapse, to preserve abstinence or to reduce harm, but also how to handle a relapse if occurred, such that further relapses can be prevented (17, 28). Possibly, the model is specifically beneficial when it comes to IGD as the confrontation with triggers is particularly frequent, considering young people’s constant access to gaming via smart phones, tablets and computers.

Interestingly, both the control and the treatment group improved significantly regarding mean GASA score from baseline to follow-up. The findings on the natural course of IGD differ across studies (29). Gentile et al. showed that 84% of the pathological gamers, in a secondary school setting, were still pathological gamers 2 years later (30). Another study, also conducted on a sample of secondary school students, showed that 50% of the addicted gamers were still addicted 1 year later (31) while Krossbakken et al. reported on a three-year stability of 35%, among a representative sample of Norwegian 17-year-olds (3).

The fact that this trial also showed a significant improvement regarding IGD symptomatology in the control group could reflect the self-healing nature of the condition, but it could also be a consequence of the fact that the control group did receive some form of psychiatric care. Possibly, their improvement was a positive side effect of adequate care of another psychiatric comorbidity. It is evident that there is a reciprocal link between psychological distress and IGD (3) and it is therefore possible that treatment of psychiatric problems had some positive spillover effect on IGD.

The treatment group in this trial improved to a higher degree relative to the control group. Additionally, the analyses of prevalence of gaming categories showed a significant decrease of problem and addicted gamers in the treatment group but not in the control group, which possibly should be considered more clinically relevant than the change in GASA score (24, 25). The prevalence of addicted gamers dropped by 79% in the treatment group, in comparison to a drop by 47% in the control group. Comparing this treatment efficacy with findings of previous research is not entirely straightforward as comparable studies are few and the outcome measures differ. Zajac et al. summarized the research field in a systematic review published in 2020, in which they identified only four previously published RCT evaluating CBT-based treatments of IGD. Among these trials, two did not find an advantage of CBT over control (13). One of the other two reported that a mindfulness-oriented group treatment was superior to a support group, in a sample of 30 students and university employees (32). The other successful trial showed that combined CBT and bupropion was an effective treatment of IGD in 65 male adolescents with major depressive disorder (33); thus, a study carried out in a very specific population. The less successful RCTs both provided therapeutically active treatments for the control group, and both had a relatively small sample size with 28 and 24 participants, respectively (34, 35). In summary, previous comparable research is barely existent, and the findings are not entirely clear-cut.

This trial contributes with further support for CBT-based treatments of IGD, specifically RP. RP has the advantages of being a relatively short, low cost and manual-based treatment that does not place higher demands on the practitioner than the basic psychotherapeutic competence. The treatment could thus be offered outside of psychiatry, such as through primary care or school healthcare. Knowledge gaps remain, such as how the family situation and parent–child relationships can affect and might be affected by IGD treatment (20). Also, future research should address which aspects within the given treatment are effective, who benefits from treatment, in what aspects, and why.

### 4.1. Strengths and limitations

The presented findings should be considered in the light of the study’s limitations. One limitation is the fact that the treatment group showed a higher GASA score than the control group at baseline, which might impact the relative efficacy of treatment. One could argue that an individual with greater gaming problems would show a greater improvement than an individual with less pronounced problems, representing a ceiling effect (36). However, when the baseline score was controlled for, the effect of the treatment remained significant, which supports the main findings in the study.

One other potential limitation is the absence of blinding which entails a risk that the participants in the control group, and possibly also their parents, experienced disappointment when they were informed that they had been randomized to a group that would not receive gaming-specific treatment. Possibly this disappointment contributed to a reduction in improvement that might have been seen otherwise.

The fact that TAU could not be kept constant is another limitation. The interventions in the control group differed due to the diversity in the sample and TAU was not given for a particular diagnosis, but more non-specifically for each of the participants individual psychiatric problems. This is the naturalistic setting of CAP Skåne. As no specific treatment to date is provided targeting gaming behavior among adolescents within the Swedish CAP context, this methodological approach was the most reasonable for us.

One other possible limitation is the fact that GASA applies to experiences with games over the last 6 months whereas the DSM-5 criteria for IGD concern the last 12 months (6). However, GASA is developed for adolescents specifically (22) and our clinical understanding and experience of youth gaming is that 6 months of destructive gaming is enough to cause negative consequences and a need for help.

Also, measures other than GASA, and reflecting additional psychological health complaints used as secondary outcomes, would have contributed valuable information on the potential range of effects of the treatment provided.

One could argue that the fact that each of the participants was diagnosed with a psychiatric condition might affect the generalizability of the results. However, this specific circumstance could also be considered as strengthening the external validity since psychiatric comorbidity, not least ADHD, is a known feature of IGD (1). Our results show that the given treatment appears to be effective in an actual clinical setting, among individuals with psychiatric comorbidity who could be considered particularly difficult to treat.

Given the limitations mentioned, the current study is to our knowledge the largest RCT to evaluate a CBT treatment for IGD among children and adolescents, and the findings are promising.

### 4.2. Conclusion

Relapse prevention was found to be superior to TAU in terms of reduction of IGD symptoms among children and adolescents in CAP clinics. The present study adds to a research field still in its infancy with further evidence that CBT, and specifically RP can be an effective treatment for IGD among children and adolescents.

## Data availability statement

The raw data supporting the conclusions of this article will be made available by the authors, without undue reservation.

## Ethics statement

The studies involving humans were approved by the Swedish Ethical Review Authority (Ref 2019-04797, December 13, 2019). Subsequent amendments was approved (Ref 2021-05592-01, January 3, 2021; Ref 2022-01289-02, March 15, 2022). The studies were conducted in accordance with the local legislation and institutional requirements. Written informed consent for participation was not required from the participants or the participants’ legal guardians/next of kin. Written informed consent was obtained for every participant and caregivers’ consents were required for children younger than 15 years in concordance with Swedish regulations.

## Author contributions

FA: Conceptualization, Data curation, Formal analysis, Investigation, Methodology, Software, Visualization, Writing – original draft. SK: Conceptualization, Investigation, Methodology, Project administration, Supervision, Visualization, Writing – review & editing. IE: Conceptualization, Methodology, Visualization, Writing – review & editing. ST: Conceptualization, Methodology, Visualization, Writing – review & editing. LF: Conceptualization, Methodology, Visualization, Writing – review & editing. AM: Conceptualization, Methodology, Visualization, Writing – review & editing. AH: Conceptualization, Methodology, Supervision, Validation, Writing – review & editing. EC: Conceptualization, Investigation, Methodology, Project administration, Resources, Software, Supervision, Visualization, Writing – review & editing.
